# Construction and validation of a prognostic model for predicting overall survival of primary adrenal malignant tumor patients: A population-based study with 1,080 patients

**DOI:** 10.3389/fsurg.2022.1025213

**Published:** 2022-10-24

**Authors:** Wenhao Xie, Yida Zhang, Runfu Cao

**Affiliations:** Department of Urology, The First Affiliated Hospital of Nanchang University, Nanchang, China

**Keywords:** adrenal malignant tumor, SEER, prognosis, overall survival, nomogram, construct, decision curve, validate

## Abstract

**Objective:**

Primary adrenal malignant tumor is rare. The factors affecting the prognosis remain poorly defined. This study targeted to construct and corroborate a model for predicting the overall survival of adrenal malignant tumor patients.

**Methods:**

We investigated the SEER database for patients with primary adrenal malignant tumor. 1,080 patients were divided into a construction cohort (*n* = 756) and a validation cohort (*n* = 324), randomly. The prognostic factors for overall survival were evaluated using univariate and multivariate Cox analyses. The nomogram was constructed and then validated with C-index, calibration curve, time-dependent ROC curve, and decision curve analysis in both cohorts. Then we divided the patients into 3 different risk groups according to the total points of the nomogram and analyzed their survival status by Kaplan-Meier curve with log-rank test.

**Results:**

The baseline characteristics of these two cohorts were not statistically different (*P* > 0.05). Using univariate and multivariate Cox analyses, 5 variables, including age, tumor size, histological type, tumor stage, and surgery of primary site, were distinguished as prognostic factors (*P* < 0.05). Based on these variables, we constructed a nomogram to predict the 3- year, 5- year, and 10-year overall survival. The C-indexes were 0.780 (0.760–0.800) in the construction cohort and 0.780 (0.751–0.809) in the validation cohort. In both cohorts, the AUC reached a fairly high level at all time points. The internal and external calibration curves and ROC analysis showed outstanding accuracy and discrimination. The decision curves indicated excellent clinical usefulness. The best cut-off values for the total points of the nomogram were 165.4 and 243.1, and the prognosis was significantly different for the three different risk groups (*P* < 0.001).

**Conclusion:**

We successfully constructed a model to predict the overall survival of primary adrenal malignant tumor patients. This model was validated to perform brilliantly internally and externally, which can assist us in individualized clinical management.

## Introduction

Worldwide, primary adrenal malignant tumor is rare among the population (1, 2). As we know, the most common histological types are adrenocortical carcinoma (ACC) and pheochromocytoma (PHEO) (3, 4). Other types of adrenal malignant tumors are sporadic (5, 6). ACC is the major adrenal malignant tumor in clinical work, and even so, its incidence is low, with an annual incidence estimated to be only 2 in a million (7, 8). ACC has an ominous prognosis, with a five-year survival rate ranging from 16 to 38% and a median overall survival of about 3–4 years (9, 10). PHEO is currently believed to harbor malignant potential, with an incidence of 3 cases/million /year (11, 12). About 5%–20% of PHEO will exhibit metastatic behavior (13, 14). The Mayo Clinic reported a 5-year survival rate of 36%, with most deaths occurring within 3 years of detection of metastasis (15). Adrenalectomy is the mainstay treatment (16). For PHEO, total adrenalectomy seems to be more recommended in the guidelines, but partial resection has not been discontinued, either (17–19). Mitotane is the only oral drug officially approved for ACC (20, 21). Radiotherapy and chemotherapy, are possible choices for advanced and recurrent patients, but the improvement in overall survival is controversial (22–24). As mentioned above, although adrenal malignant tumors are relatively rare, they have a poor prognosis and deserve our attention. Due to the rarity of patients with adrenal malignant tumor, there are few researches on the prognostic factors (25–27). Previous researches mainly focused on only one histological type and included few cases and variables (28, 29). Published literatures did not have risk classification system for patients with adrenal malignant tumor, either. Hence, when patients are diagnosed with adrenal malignant tumor, we can rarely predict their overall survival in individual.

Therefore, the interest of this study is to predict the overall survival of each individual based on the patient clinical information and treatment measures. To achieve this objective, we defined the prognostic factors for adrenal malignant tumor. So that we can visualized the probability of overall survival for each patient in a novel form of nomogram individually. Then we validated this model internally and externally. We also created a risk classification system that will grade patients according to their risk of overall survival. Thereby, we believe we can assist with personalized clinical decision and surveillance in clinical work.

## Patients and methods

### Patients

**Figure 1:**
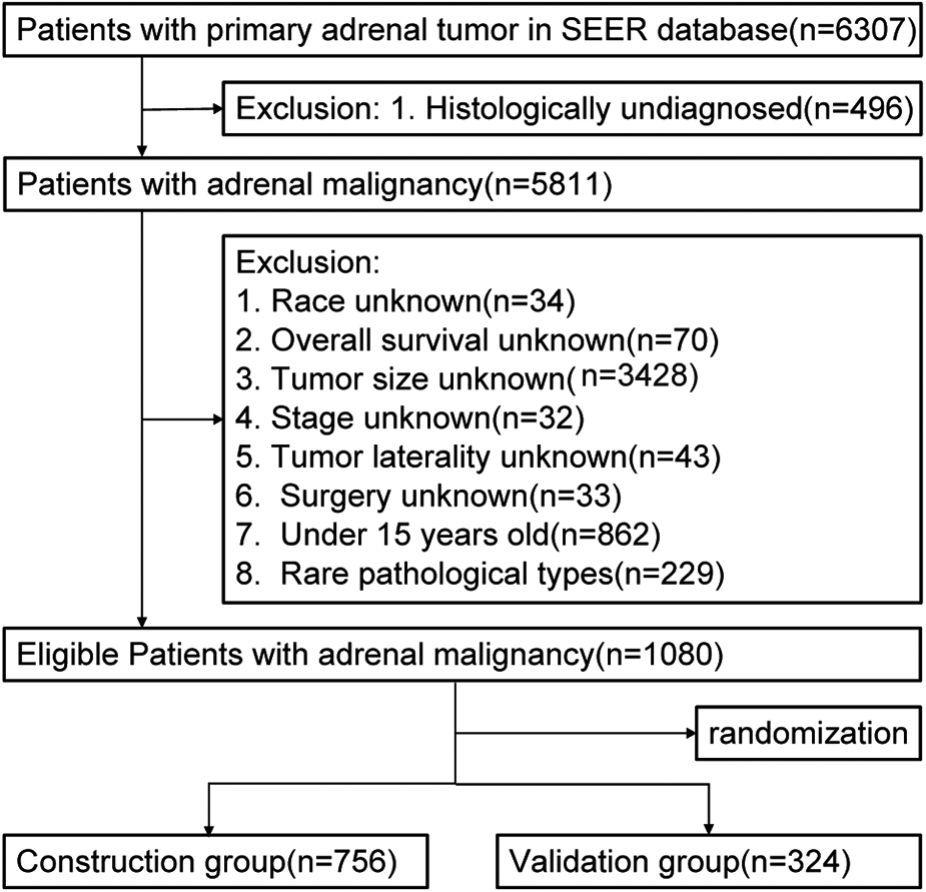
Flow chart of selection.

Our study extracted the records of patients initially diagnosed with primary adrenal malignant tumor from 5 databases in the Surveillance, Epidemiology, and End Results (SEER) database (Figure 1). The deadline for statistics was November 2019. We retrieved 6,307 patient cases primordially. Exclusion criteria included the following: the patients whose tumor size, surgical method, race, tumor laterality, histological differentiation, and overall survival were unknown; the patients below 15 years old (30); the patients with rare histological types (31). Ultimately, 1,080 patient cases were eligible to be enrolled. All data in this study were publicly available and deidentified. Therefore, ethical approval and informed consent were not needed.

Statistical variables for each patient included: patient ID, age, sex, race, tumor size, laterality of tumor, tumor stage, histologic type, surgery of primary site, radiotherapy, chemotherapy, survival months, and vital status. The race included white, black, and other races. Tumor size was defined as the maximal diameter of the tumor in millimeters. Laterality of tumor means the side the tumor originated from. Based on the “SEER Combined Summary Stage 2000 (2004+)”, the stage of tumor was restaged as localized only (I), regional lymph nodes involved only (II), regional by direct extension only (III), regional by both direct extension and lymph node involvement (IV) and distant site(s)/node(s) involved (V) (32, 33). The histologic type contained ACC and PHEO. Surgery of the primary site consisted no surgery, partial adrenalectomy and total adrenalectomy. Radiotherapy and chemotherapy were divided into done or no/unknown. In this study, the endpoint was set as overall survival.

### Statistical analysis methods

In a 7:3 split ratio, we split 1,080 eligible patients into the construction and validation cohorts, randomly. Unless indicated otherwise, the categorical variables were expressed as numbers and percentages (*N*, %), while the continuous variables were reported as median (range). To compare the baseline characteristics data between the construction and validation cohorts, the continuous variables were analyzed by Mann–Whitney *U* test, and the categorical variables were analyzed by Chi-square test or Fisher exact test.

We applied the backward procedure with the Akaike information criterion to select the variables. Univariate Cox and multivariate Cox analysis were executed to assess variables' ability to predict OS. The hazard ratio (HR) and 95% CI were used to show the overall risks. The selected variables which reached statistical significance were incorporated to construct the nomogram. The nomogram displayed the rates of 3-year, 5-year, and 10-year overall survival of primary adrenal malignant tumor patients.

Then we validated the nomogram internally and externally. We employed calibration curve, concordance index (C-index), time-dependent ROC analysis, and area under curve (AUC) to evaluate the predictive accuracy and discrimination ability. And the clinical utility was accessed by the decision curve. We used x-tile to calculate the best cut-off values for the points of the nomogram, and classified patients with different scores into low-risk, middle-risk, and high-risk groups. We then compared the survival status of the different groups using Kaplan-Meier method with log-rank test. All statistical analyses were proceeded using R software 4.2.0 and IBM SPSS Statistics 26 and x-tile software.

## Results

### Patient baseline characteristics

In the study, we enrolled 756 patients in the construction cohort and 324 patients in the validation cohort finally. The baseline characteristics of these two cohorts were listed. (Table 1). There was no statistical difference among all baseline characteristics of these two cohorts (*p* > 0.05). For all patients, females, the white race were the majority, and the median patient age was 56 years (range, 15–91 years). The median tumor size was 100 mm (range, 0–800 mm) in the construction and 96.5 mm (range, 0–345 mm) in the validation cohort. More tumors were located on the left. In the construction and validation cohorts, ACC accounted for 81.6% and 79% of the total cases, correspondingly. Most tumors were distant sites/nodes involved. Other tumors were mainly *in situ*, with the least regional invasion. About one-third of the patients performed surgery on the primary site and received chemotherapy, and bits of patients received radiotherapy. 36.1% of the construction cohort patients and 33.0% of the validation cohort patients were still alive. In these two cohorts, the median survival months were 28 (range, 0–165) and 22 (range, 0–166), separately.

**Table 1:** Characteristics of patients with primary adrenal malignant tumor in the construction cohort and validation cohort.

Characteristic	Total *N* (%)	Construction cohort *N* (%)	Validation cohort *N* (%)	*P*
	1,080 (100)	756 (70)	324 (30)	
Age	56 (15–91)	56 (15–90)	56 (15–91)	0.994
Sex				0.403
Male	446 (41.3)	306 (40.5)	140 (43.2)	
Female	634 (58.7)	450 (59.5)	184 (58.7)	
Race				0.957
White	888 (82.2)	621 (82.1)	267 (82.4)	
Black	115 (10.6)	80 (10.6)	35 (10.8)	
Others	77 (7.1)	55 (7.3)	22 (6.8)	
Size (mm)	100 (0–800)	100 (0–800)	96.5 (0–345)	0.113
Laterality				0.140
Left	537 (53.1)	390 (51.6)	183 (56.5)	
Right	507 (46.9)	366 (48.4)	141 (43.5)	
Tumor stage				0.098
I	404 (42.0)	332 (43.9)	122 (37.7)	
II	20 (1.9)	14 (1.9)	6 (1.9)	
III	197 (18.2)	143 (18.9)	54 (16.7)	
IV	26 (2.4)	15 (2.0)	11 (3.4)	
V	383 (35.5)	252 (33.3)	131 (40.4)	
Histological type				0.320
Adrenocortical carcinomas	873 (80.8)	617 (81.6)	256 (79.0)	
Pheochromocytoma	207 (19.2)	139 (18.4)	68 (21.0)	
Surgery of primary site				0.902
Not done	228 (21.1)	151 (20.0)	77 (23.8)	
Partial adrenalectomy	235 (21.8)	171 (22.6)	64 (19.8)	
Total adrenalectomy	617 (57.1)	434 (57.4)	183 (56.4)	
Radiotherapy				0.988
No/unknown	922 (22.3)	339 (22.3)	145 (22.3)	
Done	158 (77.7)	1,181 (77.7)	506 (77.7)	
Chemotherapy				0.910
No/unknown	703 (85.4)	646 (85.4)	276 (85.2)	
Done	337 (14.6)	110 (14.6)	48 (14.8)	
Status				0.330
Alive	380 (35.2)	273 (36.1)	107 (33.0)	
Dead	700 (64.8)	483 (63.9)	217 (67.0)	
Survival months	27 (0–166)	28 (0–165)	22 (0–166)	0.101

### Survival analyses

With the Akaike information criterion, the backward selection procedure was applied to select the prognostic predictors for OS in the construction cohort (Table 2). Age (*P* < 0.001), race (*P* = 0.005), tumor size (*P* < 0.001), histology type (*P* < 0.001), tumor stage (*P* < 0.001), chemotherapy (*P* < 0.001), and surgery of primary site (*P* < 0.001) were statistically significant variables in the univariate analysis. Then these variables were subsumed into the multivariate Cox analysis. In the multivariate Cox analysis, 5variables including age [HR: 1.019 (1.013–1.026); *P* < 0.001], tumor size [HR: 1.001 (1.000–1.002); *P* < 0.001], histological type [HR: 0.328 (0.238–0.452); *P* < 0.001], tumor stage (*P* < 0.001), and surgery of primary site (*P* < 0.001), were ascertained as independent prognostic factors. As the tumor stage progresses, the risk of all-cause death increases. ACC has the worst prognosis. The C-indexes were 0.780 (0.760–0.800) and 0.780 (0.751–0.809) in the construction and the validation cohorts, respectively.

**Table 2:** Multivariate Cox analysis and univariate Cox analysis of overall survival in the construction cohort.

	Univariate analyses		Multivariate analyses	
	HR (95%CI)	*P*	HR (95%CI)	*P*
Sex (female vs. male)	0.874 (0.730–1.048)	0.146		
Age	1.021 (1.015–1.027)	<0.001*	1.019 (1.013–1.026)	<0.001*
Race (Ref. = white)		0.005		0.633
Black	0.578 (0.414–0.806)	0.001	0.857 (0.609–1.250)	0.374
Others	0.960 (0.680–1.356)	0.818	0.926 (0.652–1.315)	0.667
Size	1.003 (1.002–1.004)	<0.001*	1.002 (1.000–1.003)	0.040*
Laterality (right vs. left)	1.034 (0.865–1.236)	0.717		
Tumor stage (Ref =I)		<0.001*		<0.001*
II	1.407 (0.622–3.184)	0.413	1.558 (0.684–3.549)	0.291*
III	1.844 (1.422–2.393)	<0.001*	1.722 (1.324–2.240)	<0.001*
IV	4.755 (2.743–8.243)	<0.001*	5.377 (3.087–9.366)	<0.001*
V	5.060 (4.081–6.274)	<0.001*	3.715 (2.874–4.802)	<0.001*
Histological type (PHEO vs. ACC)	0.308 (0.228–0.415)	<0.001*	0.328 (0.238–0.452)	<0.001*
Surgery of the primary site (Ref. = total adrenalectomy		<0.001*		<0.001*
Partial adrenalectomy	0.786 (0.616–1.001)	0.051	0.983 (0.768–1.259)	0.667
Not done	4.161 (3.363–5.148)	<0.001*	2.626 (2.036–3.387)	<0.001*
Radiotherapy (no/unknown vs. done)	1.027 (0.793–1.330)	0.838		
Chemotherapy (no/unknown vs. done)	0.621 (0.516–0.748)	<0.001*	1.195 (0.966–1.479)	0.100

* *P* < 0.05; PHEO, pheochromocytoma; ACC, adrenocortical carcinomas.

### Nomogram construction and validation

**Figure 2:**
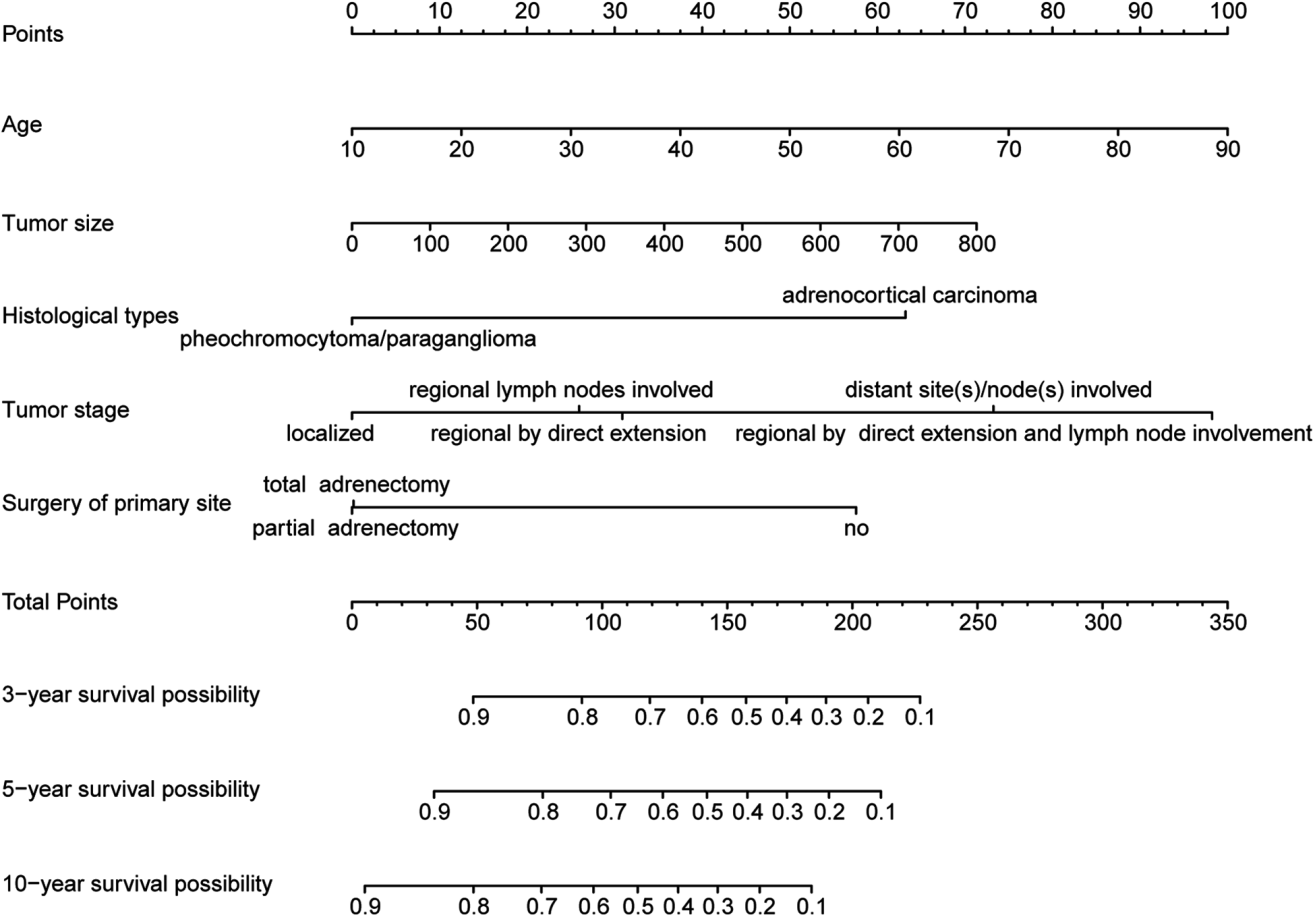
Nomogram for predicting 3-year, 5-year and 10-year overall survival of primary adrenal malignant tumor patients in the construction cohort.

Those above-mentioned 5 independent prognostic factors, including sex, age, tumor size, tumor stage, and surgery of the primary site, were incorporated to establish the nomogram in the construction cohort (Figure 2). With the help of this nomogram, we visually predicted the 3-year, 5-year, and 10-year OS of patients with adrenal malignant tumors. As shown in these figures, the prognosis was mainly influenced by age and tumor stage. Tumor size and histological type showed moderate impacts on the prognosis. Whether surgery or not may have a relatively small impact on prognosis, which cannot be ignored, either.

**Figure 3:**
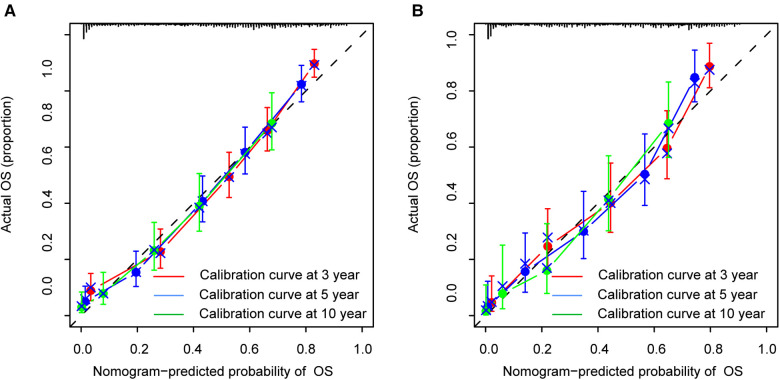
Calibration curves at 3-year, 5-year and 10-year points in the construction cohort (**A**) and in the validation cohort (**B**).

**Figure 4:**
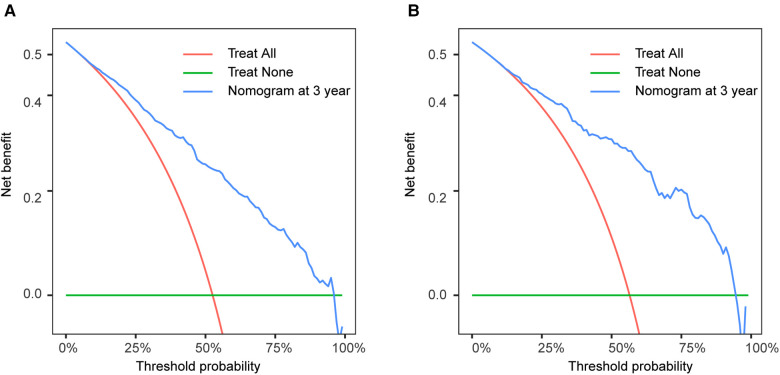
Decision curve analysis at 3-year point in the construction cohort (**A**) and in the validation cohort (**B**).

**Figure 5:**
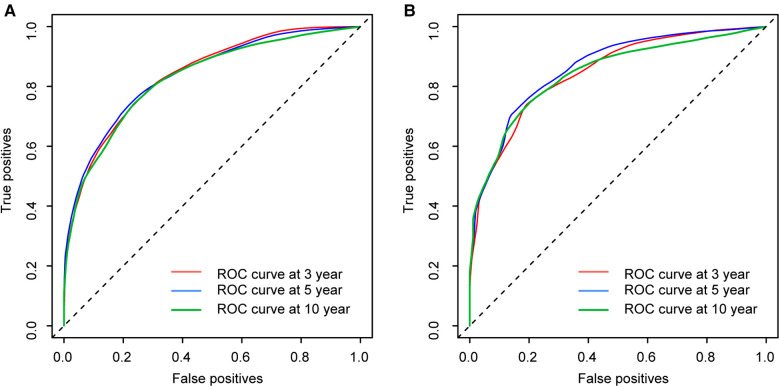
Time-dependent ROC curves at 3-year, 5-year and 10-year points in the construction cohort (**A**) and in the validation cohort (**B**).

**Figure 6:**
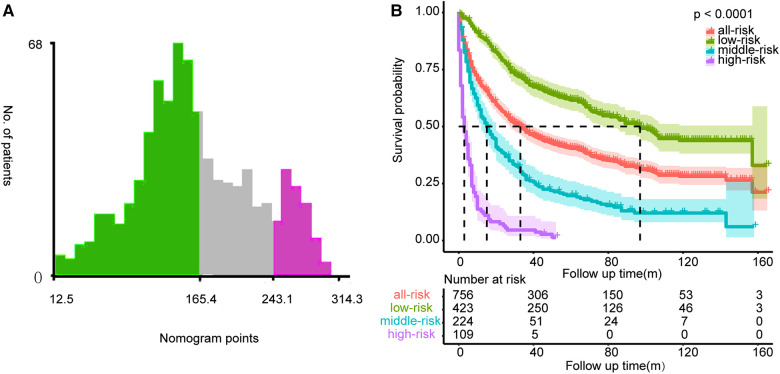
The best cut-off values of the total points of the nomogram (**A**) and Kaplan-Meier curves with log-rank test for the low-risk, middle-risk, and high-risk groups (**B**).

The calibration curves certificated brilliant agreement between the actual and predicted survival at 3 -year, 5-year, and 10-year points, which verified the outstanding accuracy (Figure 3). The AUCs at 3-year, 5-year, and 10-year OS have reached 0.842 (0.813–0.870), 0.840 (0.809–0.871), and 0.834 (0.779–0.889) in the construction cohort, and 0.857 (0.813–0.899), 0.8736 (0.830–0.917), and 0.856 (0.781–0.931) in the validation cohort, respectively. The decision curves proved positive net benefits in predicting 3-year OS in these two cohorts, thus indicating the favorable clinical utility (Figure 4). In both cohorts, the time-dependent ROC analysis demonstrated good discrimination at all time points (Figure 5). We found the best cut-off values of the total points in the nomogram were 165.4 and 243.1, using the X-tile (Figure 6). The Kaplan-Meier method and log-rank test (*P* < 0.001) demonstrated statistically significant differences in overall survival across the low-risk (12.5–165.4), middle-risk (165.4–243.1), and high-risk (243.1–314.3) groups. This risk classification system can clearly distinguish between patients with different risks.

## Discussion

Our present study defined 5 prognostic factors, including age, sex, tumor size, histological type, and surgery of primary site. With the nomogram, we clearly visualized showed the overall survival of each patient. The risk classification system classified patients into low-risk, middle-risk and high-risk groups and was well differentiated. Moreover, both the C-index, calibration curve, decision curve, time-dependent ROC curve, and AUC performed very well in internal, and external validation at 3, 5, and 10 years points. These provided ample evidence of the excellent accuracy, discrimination and clinical validity of our model. Interestingly, these curves seemed to perform better in the validation cohort than in the construction cohort. This may be because the initial data were randomly grouped, proving our model is excellent from the side. This model elicited several meaningful findings as followed. We know that age and tumor stage have the greatest impact on overall survival, followed by tumor size, and that histological type and surgical procedure have a relatively small, but not insignificant impact on prognosis. Specifically, the older the age at diagnosis or the larger the size of the tumor the significantly worse the prognosis of the patient (34, 35). For the tumor stage, if the tumor has infiltrated the surrounding organs or has metastasized distantly, the patient’s expected overall survival is significantly shorter, with distant metastasis having the worst prognosis, which is also in line with our general impression. However, patients who have only local lymph node involved do not have a significantly different prognosis from that of *in situ* tumors. Patients with PHEO have a significantly better prognosis than patients with ACC. Surgery at the primary site can significantly improve the prognosis of patients. A noteworthy point is that partial adrenalectomy does not show a significant difference from total adrenalectomy. In univariate analysis, race and chemotherapy appeared to be prognostic factors, but after multifactorial analysis corrected for several variables, we knew that chemotherapy and race did not affect prognosis (36, 37).

To our best knowledge, our research represents the largest cohort involving primary adrenal malignant tumor patients, totaling 1,080 patients (38, 39). Therefore, this study is pretty representative. There are some researches have also focused on the prognosis of adrenal malignant tumors. The study by Kirellos Said Abbas et al. advocated that gender is a prognostic factor for anaplastic tumors (40). In contrast, our study is more supportive that gender does not have a significant effect on the prognosis of patients. Unlike the research by Junjiong Zheng et al., we excluded pediatric patients below 15 years old, when including the age variable (41). This is because pediatric patients may have a different course because of pathology and patients may often be affected by genetic disorders and therefore they show different clinical and prognostic characteristics than the adult population (42, 43). Our study demonstrates that in adult adrenal patients age is the largest factor affecting prognosis instead of the smaller effect on children in their research (44, 45). As described in many reviews, surgery of the adrenal gland is the current common standard for adrenal tumors (46, 47). Our study discloses that patient who undergoes surgery has a significantly better prognosis, but total adrenalectomy does not have a significantly better prognosis compared to partial adrenalectomy, which is supported by the study of Silvinato et al. and other researches laterally (48–50). Accordingly, partial adrenalectomy may be considered when clinical specifications are followed (51). Distinct from most previous studies, our histological type included both ACC originating from the adrenal cortex and PHEO from the adrenal medulla (52, 53). The reason for including two heterogeneous histological types in this study, in addition to validating previous findings that PHEO has a better prognosis than ACC, was mainly because we wanted to make a simple-to-use model for clinical workers. In the clinical workup, if patients suffer ACC, then they are likely to have one of the above two histological types, so that we can easily get a prediction of the overall survival of the patient using our nomogram directly.

Inevitably, there are several shortages in our study. We know that PHEO is usually influenced by family history and genetic factors, but our study data were derived from the SEER data, so the information on family history was unfortunately absent. Molecular biology as well as genetic parties and information on hormone secretion specific to adrenal malignant tumor patients were also not available from the database (54). We additionally need prospective clinical trials to verify our study.

## Conclusion

In conclusion, we identified the independent prognostic factors for OS of primary adrenal malignant tumor patients. The proposed nomogram performed well on internal and external validation, showing satisfying accuracy, discrimination, and clinical utility. This prediction model can be used as an independent tool to assess the prognosis and guide personalized clinical management of primary adrenal malignant tumor patients.

## Data Availability

Publicly available datasets were analyzed in this study. This data can be found here: https://seer.cancer.gov/data/.
